# Exergames Inherently Contain Cognitive Elements as Indicated by Cortical Processing

**DOI:** 10.3389/fnbeh.2018.00102

**Published:** 2018-05-18

**Authors:** Phillipp Anders, Tim Lehmann, Helen Müller, Karoline B. Grønvik, Nina Skjæret-Maroni, Jochen Baumeister, Beatrix Vereijken

**Affiliations:** ^1^Department of Neuromedicine and Movement Science, Norwegian University of Science and Technology (NTNU), Trondheim, Norway; ^2^Exercise Neuroscience & Health Lab, Institute of Health, Nutrition and Sport Sciences, University of Flensburg, Flensburg, Germany; ^3^Exercise Science and Neuroscience, Department of Exercise & Health, Faculty of Science, Paderborn University, Paderborn, Germany

**Keywords:** exergaming, balance, EEG, theta, alpha, cognition

## Abstract

Exergames are increasingly used to train both physical and cognitive functioning, but direct evidence whether and how exergames affect cortical activity is lacking. Although portable electroencephalography (EEG) can be used while exergaming, it is unknown whether brain activity will be obscured by movement artifacts. The aims of this study were to assess whether electrophysiological measurements during exergaming are feasible and if so, whether cortical activity changes with additional cognitive elements. Twenty-four young adults performed self-paced sideways leaning movements, followed by two blocks of exergaming in which participants completed a puzzle by leaning left or right to select the correct piece. At the easy level, only the correct piece was shown, while two pieces were presented at the choice level. Brain activity was recorded using a 64-channel passive EEG system. After filtering, an adaptive mixture independent component analysis identified the spatio-temporal sources of brain activity. Results showed that it is feasible to record brain activity in young adults while playing exergames. Furthermore, five spatially different clusters were identified located frontal, bilateral central, and bilateral parietal. The frontal cluster had significantly higher theta power in the exergaming condition with choice compared to self-paced leaning movements and exergaming without choice, while both central clusters showed a significant increase in absolute alpha-2 power in the exergaming conditions compared to the self-paced movements. This is the first study to show that it is feasible to record brain activity while exergaming. Furthermore, results indicated that even a simple exergame without explicit cognitive demands inherently requires cognitive processing. These results pave the way for studying brain activity during various exergames in different populations to help improve their effective use in rehabilitation settings.

## Introduction

Exergames are videogames that require bodily movements by the user in order to play the game (Brox et al., 2011). Even though most commercial exergames are primarily developed for entertainment purposes (Zyda, 2005), exergames have in recent years been considered valuable to encourage participation in exercise, as well as to improve adherence to exercise and rehabilitation tasks (Burke et al., 2009; Skjæret et al., 2016). Exergames have rapidly gained popularity in all age groups the last decade (e.g., Mellecker et al., 2013), and have shown positive effects on increased physical activity in general (e.g., Höchsmann et al., 2016; Rhodes et al., 2017), as well as in specific rehabilitation settings (e.g., Laver et al., 2012; Baltaci et al., 2013). Several studies have concluded that exergames can be beneficial when used as an adjunct to, or even instead of, usual care, as exergames are generally found to be as effective as—or more effective than—traditional exercise programs, with generally no reported negative effects (Skjæret et al., 2016).

Exergames do not only address physical activity, but have the potential to influence players’ cognitive abilities as well through dual tasks, decision making tasks and discrimination tasks (Zelinski and Reyes, 2009; Anguera et al., 2013). As many of these additional tasks require multiple cognitive processes, exergames may have advantages over separate physical or cognitive interventions, as simultaneous physical activities with decision-making opportunities may be essential to maximize synergistic benefits (Basak et al., 2008; Yan and Zhou, 2009; Anderson-Hanley et al., 2012; Kraft, 2012). However, most of these positions have not yet been substantiated with direct empirical support. Most studies either used cognitive tests as proxy measures for cognitive processing rather than directly measuring brain activity during exergaming, or demonstrated changes in cortical activation in pre-post exergame intervention designs. For example, Eggenberger et al. (2016) found significantly reduced oxygenation in the prefrontal cortex after 8 weeks of interactive cognitive-motor exergame training, as examined with functional near infrared spectroscopy. Furthermore, Anguera et al. (2013) used electroencephalography (EEG) to quantify cortical processing before and after video game training, and demonstrated enhanced frontal theta power and fronto-parietal theta coherence as indicators for improved cognitive processes. While these studies indirectly addressed cognitive processing related to exergaming, only Baumeister et al. (2010) directly assessed brain activity during exergaming in a virtual golf-putting environment. Their EEG results revealed increased frontal theta and decreased parietal alpha-2 power during virtual putting compared to a resting period. Collectively, these electrophysiological approaches to cognitive elements of exergaming indicate alterations of cortical activation, suggesting changes in cognitive control during and after gameplay, but there is still a lack of knowledge regarding cortical processing during exergaming. Furthermore, no study has investigated whether exergames inherently require different cortical processing compared to performing similar movements without exergame guidance, and whether additional cognitive elements in an exergame further increase demands on executive functioning. Gaining more knowledge about whether and how cognitive tasks in exergames influence cortical activity could elucidate underlying neurobiological mechanisms (Stanmore et al., 2017), thereby allowing more effective use of exergames in exercise and rehabilitation settings. Therefore, the aims of the current study were to investigate whether it is feasible to measure brain activity during gameplay using EEG and if so, whether exergames inherently require cortical processing, and whether increased cognitive demand in the exergame further changes brain activity.

To address these aims, we measured cortical activity using a portable EEG system in young, healthy participants. In order to reduce movement artifacts in the EEG signals as much as possible, we chose a puzzle exergame that is played by performing simple sideways leaning movements. Furthermore, the puzzle exergame could be played with and without an additional cognitive choice task. We hypothesized that even this simple exergame would require increased cortical processing compared to performing similar movements without exergame guidance, as indicated by increased frontal theta with concomitant changes in alpha-2 activity (Sauseng et al., 2005). Similar to their experiment on visuospatial working memory tasks, participants in the current study would need to mentally manipulate a picture in order to make a correct choice. Furthermore, we hypothesized that the addition of a simple choice task would further increase cortical processing, as reflected by increasing frontal theta and decreasing parieto-central alpha-2 (Gevins et al., 1997). Activity in the alpha band was shown to be inversely related to task difficulty in order to allocate more resources to the task performance. Both Gevins et al. (1997) and Sauseng et al. (2005) described theta and alpha as neurophysiological indicators of cognitive processing related to working memory demands, with increased cortical processing related to increased frontal theta power and inversely related to alpha power in parietal areas of the cortex.

## Materials and Methods

### Participants

As this study addressed the feasibility of recording EEG while exergaming, a convenience sample of young participants was chosen. Twenty-four injury-free young adults (12 of each gender; age: 24.6 ± 2.1 years, height: 175 ± 10 cm, weight: 74.8 ± 11.8 kg) provided written consent to participate in this experimental study at the Norwegian University of Science and Technology in Trondheim, Norway. To be included, participants had to be between 20 years and 30 years old with no history of injuries or surgeries to the lower extremity and/or back within the last 6 months, no balance problems, and no neurological disorders that could affect postural control.

All participants indicated that they were physically active for at least 2–3 times a week. The majority (18 participants) described their physical activity as “quite strenuous,” with a duration of 30–90 min per session (20 participants).

This study was carried out in accordance with the recommendations of the Regional Committees for Medical and Health Research Ethics, Norway. The protocol was approved by the Regional Committees for Medical and Health Research Ethics, Norway. All subjects gave written informed consent in accordance with the Declaration of Helsinki.

### Procedure

**Figure 1:**
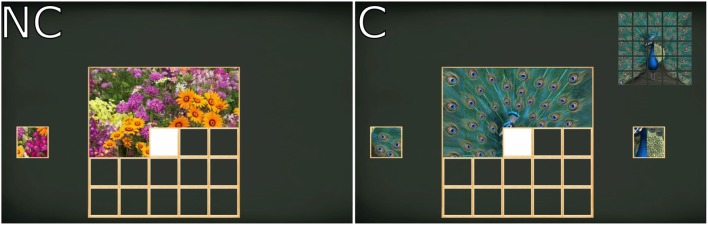
Screen images of the “Puzzle” exergame. Left panel: no-choice condition (NC) presents only the correct puzzle piece to the left or right; Right panel: choice condition (C) presents two puzzle pieces simultaneously.

We recorded EEG continuously throughout the entire protocol, starting with participants seated for 3 min, followed by 3 min of self-paced leaning sideways (SP) with feet hip-wide apart. Participants were instructed on how to perform SP before the start of the trial, without guidance or feedback during the trial. After a 2-min break, participants played a commercially available exergame («Puzzle», SilverFit, Netherlands). The aim of the exergame was to complete a screen-based 5-by-5-puzzle in sequential order. Puzzle pieces were selected by leaning sideways in the direction of the desired puzzle piece. This simple exergame was chosen as it can be played without vigorous movements, so as to mitigate the risk for movement artifacts in the EEG signal. Each participant played the exergame in two conditions (“no-choice condition” NC and “choice condition” C) and with two different target pictures as shown in Figure 1, counterbalanced across participants. In NC, participants were presented with one puzzle piece, which was selected by leaning in the corresponding direction. In C, participants had to choose between two puzzle pieces and lean in the direction corresponding to the correct piece. Each participant played two sets, each consisting of ten exergames for NC or C. After playing all 20 exergames, another seated EEG baseline was recorded for 2 min followed by 1 min of self-paced leaning. In a preliminary examination the day before data collection, participants were familiarized with the laboratory environment and the EEG equipment. Furthermore, head circumferences were measured in order to choose the proper EEG cap size.

### Performance Measures

To check whether participants made similar leaning movements in the different conditions, two force platforms (Kistler, type 9286A, Winterthur, CH) with a measurement frequency of 100 Hz were used to record ground reaction forces. The force platforms were located 2.5 m in front of the exergame screen. The mean of the medio-lateral center of pressure (COP) amplitude peaks for each sideways lean as well as the overall COP velocity were calculated. Due to technical issues with the force platforms, COP trajectories could not be calculated for four trials.

### EEG Recordings and Analysis

Cortical activity was recorded continuously from 64 Ag/AgCl passive electrodes, using an elastic cap (QuikCap, Compumedics Neuroscan, Charlotte, NC, USA), with electrodes placed according to the international 10–20 electrode placement standard (Klem et al., 1999) and a standard reference electrode positioned between CZ and CPZ. Electrode impedance was reduced to <10 kΩ to ensure an appropriate signal-to-noise ratio. EEG data was amplified with an analog amplifier (SynAmps RT, Compumedics Neuroscan, Charlotte, NC, USA), which was placed in a small backpack in order to reduce mechanical stress in the cables and to allow mobility during data collection. The analog EEG signal was digitized using a 24-bit analog-to-digital converter (SynAmps RT, Compumedics Neuroscan, Charlotte, NC, USA) and subsequently recorded using Scan 4.5 (Compumedics Neuroscan, Charlotte, NC, USA) with a sample frequency of 1 kHz.

The EEGLAB 14.0.0b (Delorme and Makeig, 2004) toolbox for MATLAB (Mathworks Inc., Natick, MA, USA) was used for processing the acquired EEG data. The digitized EEG signal was band limited between 1 Hz and 100 Hz. The resulting digital signal was down sampled to 250 Hz after filtering using the CleanLine plugin (Mullen, 2012) to remove line noise and applying a finite impulse response filter with a band-pass between 2 Hz and 30 Hz. Any channels contaminated by excessive noise or major non-stereotypical artifacts were identified and manually deleted. EEG data was then re-referenced to common average.

Non-stereotypical artifacts were removed by visual inspection of the continuous EEG signal. Due to extensive artifact contamination, EEG data from two participants was excluded from further analysis. For the remaining participants, 47%–55% of the EEG data remained in each of the three conditions for further processing. Given the number and length of the trials, this was sufficient data to decompose the spatial-temporal sources (Onton and Makeig, 2006). Spatio-temporal features of the remaining participants were extracted using an adaptive mixture independent component analysis (AMICA; Palmer et al., 2006, 2008) on the entire dataset, resulting in spatially static and maximally independent components (Makeig et al., 1996). A heuristic approach described by Onton and Makeig (2006) was used to distinguish between functional and stereotypical artifacts.

Based on the results of the AMICA decompositions, a four-shell spherical head model (Kavanagh et al., 1978) included in the DIPFIT function (Oostenveld and Oostendorp, 2002) of EEGLAB was used to locate equivalent dipole locations of independent components. The resulting dipoles across all participants were clustered using a k-means algorithm with a preset for five clusters. Dipoles were assigned to a cluster if they were within two standard deviations of the respective cluster. Dipoles with a residual variance larger than 16% were excluded.

The absolute power of the EEG signal was calculated as area under the curve for each condition and cluster (Pivik et al., 1993) in the* a priori* defined frequency bands: theta (4–7 Hz) for the frontal cluster, as well as alpha-2 (10–12 Hz) for both central and parietal clusters.

### Statistics

All statistical analyses were performed using R 3.4.2 (R Development Core Team, 2017). Differences between SP, NC, and C were analyzed using one-way repeated measures ANOVAs on the absolute EEG power of predefined frequency bands and the performance measures, with *post hoc* paired-samples *t*-tests to follow up significant main effects. For those measures that were not normally distributed as indicated by Shapiro-Wilk’s test, namely centralL and COP amplitude, Friedman’s test was used to assess main effects, followed up by Wilcoxon’s paired signed-ranks tests. Statistical level of significance was set at *p* < 0.05.

## Results

### Performance Measures

Although the mean medio-lateral COP amplitudes were of comparable magnitude across the conditions (SP: *M* = 33.41 cm, *SD* = 7.23 cm; NC: *M* = 38.7 cm, *SD* = 4.89 cm; C: *M* = 39.98 cm, *SD* = 5.32 cm), there was a main effect of condition, $\chi_{\left(2,\;N=22\right)}^{2}$ = 11.47, *p* = 0.003. *Post hoc* Wilcoxon’s signed-ranks tests indicated that the leaning movements were significantly smaller in SP compared to C (*Z* = 3.09, *p* = 0.002) and NC (*Z* = 3.04, *p* = 0.002). COP velocity showed no significant main effect of condition (*F*_(2,36)_ = 0.07, *p* = 0.933).

### Cortical Activity

**Figure 2:**
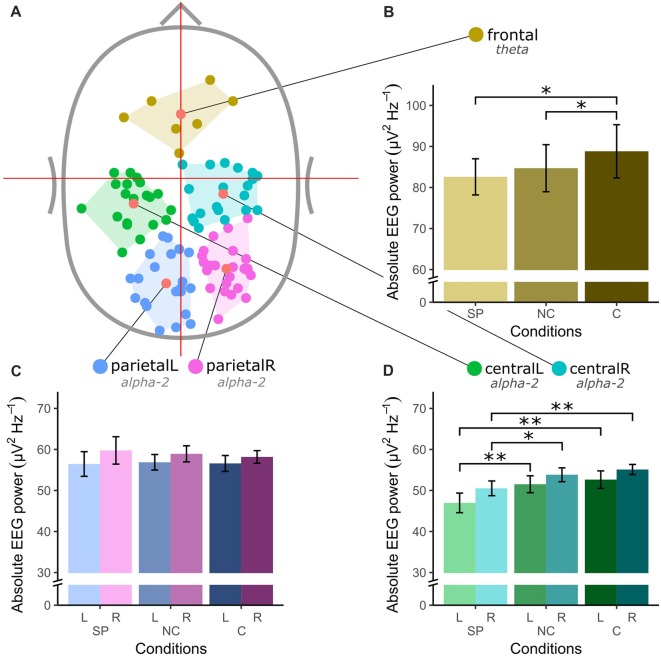
Spatial location of electroencephalography (EEG) sources and absolute EEG power in μV^2^Hz^−1^ for all clusters and conditions (SP, self-paced; NC, no-choice; C, choice) in* a priori* defined frequency bands theta (4–7 Hz) and alpha-2 (10–12 Hz). **(A)** Top-view of the identified EEG sources. **(B)** Absolute theta power in the frontal cluster. **(C)** Absolute alpha-2 power in left and right central clusters. **(D)** Absolute alpha-2 power in left and right parietal cluster. The asterisk (*) denotes a significance level of < 0.05 and the double asterisk (**) denotes a significance level of < 0.01. The error bars in **(B–D)** show the standard error.

Figure 2 shows the spatial location of EEG sources and the absolute EEG power in the respective frequency bands for all conditions and clusters. Clustering of included functional brain components revealed five robust clusters of dipoles located in the frontal (*n_IC_* = 7), bilateral central (centralL *n_IC_* = 20 and centralR *n_IC_* = 20) and bilateral parietal (parietalL *n_IC_* = 21 and parietalR *n_IC_* = 25) areas. A significant main effect for condition was found in absolute frontal theta power (*F*_(2,12)_ = 5.55, *p* = 0.02). *Post hoc* paired *t*-tests showed a significant difference between SP and C (*t*_(6)_ = 2.44, *p* = 0.05), and between NC and C (*t*_(6)_ = 2.84, *p* = 0.03). Furthermore, both clusters in the central area showed significant differences in absolute alpha-2 power (centralL $\chi_{\left(2,\;N=20\right)}^{2}$ = 9.1, *p* = 0.011; centralR *F*_(2,38)_ = 7.2, *p* = 0.003). A *post hoc* paired Wilcoxon’s signed-ranks test on centralL and a paired-samples *t*-test on centralR showed a significant difference between SP and both exergaming conditions in both clusters (centralL: SP-C *Z* = 2.91, *p* = 0.004, SP-NC *Z* = 2.91, *p* = 0.004; centralR: SP-C *t*_(19)_ = 3.11 *p* = 0.006, SP-NC *t*_(19)_ = 2.58, *p* = 0.018). No significant main effects were found for the parietal clusters.

## Discussion

The present study investigated the feasibility of measuring cortical activity in healthy young adults while playing an exergame, as well as the effect of different levels of cognitive demand on cortical activity. It was hypothesized that cortical activity in frontal, central and parietal areas of the brain would be affected differently by self-paced and exergame conditions with and without an additional choice task.

### Cortical Activity During Exergaming

The first major finding of the present study was that it was feasible to collect good quality EEG signals of cortical activity during exergaming despite participants making bodily movements. Although approximately half of the EEG signal consisted of non-stereotypical artifacts that had to be removed, the remaining signal was both quantitatively and qualitatively sufficient for finding functional brain components. Furthermore, the amplitude and velocity of the sideways leaning movements were comparable across self-paced and exergaming conditions, despite a small but significant difference in amplitude, indicating that participants’ movements were largely similar with or without exergame guidance. After cleaning and processing of the EEG data, clustering of independent components revealed five robust clusters, which were assigned to frontal, central and parietal brain areas, based on their equivalent dipole centroids. The frontal cluster was centrally located over the prefrontal cortex, while the estimated location of the equivalent mean dipoles of the central clusters was close to the lateral motor areas. In addition, the location of two lateral clusters was estimated to be in the left and right posterior-parietal cortex.

Another major finding of the present study was significantly higher absolute theta power in the frontal cluster during exergaming with choice. The task consisted of assembling a virtual puzzle by consecutively selecting matching puzzle pieces from two options displayed on the screen. This required the processing of visual information, extracting features of distinct choices, and subsequently generating a task appropriate response. The interface between these underlying perception and action circuits is responsible for temporally maintaining task relevant information and focused attention (Baddeley, 2012; Diamond, 2013). Moreover, evidence from electrophysiological studies implies that the control of cognitive processes may be indicated by theta oscillations (4–7 Hz) in the prefrontal cortex, which is known to be involved in processes of attentional control (Klimesch, 1999; Slobounov et al., 2005; Sauseng et al., 2010). Furthermore, theta activity was previously shown to be linked to rhythmic modulations of neuronal excitability in the cortex (Womelsdorf et al., 2010). As synchronized synaptic excitation is more likely to activate neuronal populations in terms of information processing, spatially and temporally dependent theta synchronization has been related to active neuronal processing for cognitive functions (Palva and Palva, 2011). Some studies previously reported increased theta synchronization with additional task requirements (Jensen and Tesche, 2002), as well as during specific selection processing of choice in goal-directed behavior (Womelsdorf et al., 2010). In line with these earlier findings, significantly higher frontal theta may indicate increased cognitive demands during exergaming with choice, compared to self-paced movement or no-choice exergaming. Since these latter conditions did not contain a choice task, higher demands on cognitive processing during exergaming with choice may explain the task-dependent increase of frontal theta in the present study.

Previous research has demonstrated that both theta and alpha-2 oscillations are involved in cognitive processes (Klimesch, 1999). While theta has been attributed to a general integrative function for the organization of cortical activity (Sauseng et al., 2005), alpha-2 has been associated with task-specific active processing or inhibition (Bazanova and Vernon, 2014), characterized by an inverse relationship between amplitude and number of neuronal populations activated (Niedermeyer and da Silva, 2005). In this regard, the present results support the notion that inhibition of task-irrelevant activity in non-essential cortical areas may facilitate cognitive processes and task performance (Klimesch et al., 2007; Palva and Palva, 2011). In particular, alpha-2 power in the central clusters demonstrated a significant increase from self-paced movement to exergaming, with no difference between the no-choice and choice exergames. The centrally located motor cortex, which contributes to movement initiation and complex motion coordination, has been reported to be involved in postural control as well (Slobounov et al., 2005, 2008). Additionally, suppressed activity within the alpha-2 frequency band in sensorimotor areas was shown to be associated with increased processing of sensory and movement-related information (Pfurtscheller and Berghold, 1989; Babiloni et al., 2014). However, the current study demonstrated alpha-2 synchronization during exergaming compared to self-paced movement. Based on reports of Jensen et al. (2002), the present results suggest that cortical activity of bilateral motor areas decreases with cognitive load. Moreover, context-dependent inhibition of the motor cortex may indicate that the generation of postural responses in exergames results from an interplay between various levels of the brain (Jacobs and Horak, 2007). Traditionally, subcortical structures like the cerebellum, basal ganglia, and brainstem have been linked to anticipatory or automatized regulation of postural control during upright stance (Nutt et al., 2011; Takakusaki, 2017). These neural structures are postulated to contribute to posture and voluntary movement through basic modifications of muscle amplitudes and patterns (Jacobs and Horak, 2007). As response latencies increase, cortical circuitries from either the prefrontal or motor cortex progressively influence subcortical pathways in order to optimize postural responses for the given environmental context (Jacobs and Horak, 2007). Thus, it may be proposed that self-paced movement with no external stimulus required active control through subcortical-cortical circuitries. Furthermore, since the dual-task conditions changed the distribution of attention resources between the cognitive and motor tasks (Fujita et al., 2016), exergaming may predominantly involve automatized subcortical processes of postural control in favor of cognitive performance.

Regarding the parietal clusters, no significant changes in alpha-2 power were found between self-paced and exergame conditions. The parietal lobe is part of a functionally interconnected sensorimotor network of motor, prefrontal and temporal cortical areas (De Waele et al., 2001). It has been suggested to play an essential role in the integration of multimodal sensory information related to voluntary movement and postural control (Varghese et al., 2015). However, consistent alpha-2 power in parietal areas may indicate that sensorimotor processing in the current postural task may not change with increased cognitive demand in a population of young adults. Furthermore, it may be hypothesized that inhibition of parieto-occipital areas may facilitate the maintenance of cognitive processes in frontal areas of the cortex (Jensen et al., 2002).

### Methodological Considerations

Although the present investigation showed that EEG measurements during exergaming are possible and show relevant findings, some methodological considerations should be highlighted. First of all, participants in the current study were healthy young adults for whom the different conditions posed little challenge. Older or less healthy populations may show stronger effects of exergaming, or indeed different effects. Furthermore, it was not possible to provide guidance or feedback during the self-paced condition without simultaneously introducing cognitive processing, which would have rendered the control condition invalid. Nonetheless, the sideways movements were quite similar across the conditions. Although the amplitude was slightly smaller in the self-paced condition, movement velocity was the same across conditions, suggesting comparable physical effort. Another potential limitation of the current research protocol is the challenging nature of EEG measurements during human movement. Although several functional clusters were identified, the presence of movement-related artifacts may have limited the number of functional brain components being decomposed and may explain the relatively small number of independent components included in the frontal cluster. With recent advances in active EEG systems and individualized head models, future studies may further elucidate cortical processing changes during exergaming.

## Conclusion

In conclusion, the present study demonstrated that even a simple exergame contains cognitive elements as indicated by task-specific cortical representation. Despite use of a passive EEG system that is sensitive to movement artifacts and the use of young adults as participants, frontal theta was found to significantly increase with increasing task demands that involve cognitive processes, such as in exergaming with a choice task. Furthermore, central alpha-2 power was significantly higher in exergame conditions compared to self-paced movement. Exergames may therefore require adjustment in the distribution of cortical resources between cognitive and motor elements in order to optimize task performance (Fujita et al., 2016). These results may provide further insight into why exergame training has been effective in improving sensorimotor processing (e.g., Gevins et al., 1997), and pave the way for follow-up research into how exergames can be used effectively to improve both cognitive and physical functions in specific populations.

## Author Contributions

PA, TL, NS-M, JB and BV contributed to the conception and design of the study. PA, HM and KG collected and processed data. PA and TL performed the statistical analyses. PA, TL, HM, KG, NS-M and BV wrote sections of the manuscript. All authors contributed to manuscript revision, and read and approved the final version.

## Conflict of Interest Statement

The authors declare that the research was conducted in the absence of any commercial or financial relationships that could be construed as a potential conflict of interest.
